# A Treatise on Ruptures

**Published:** 1893-12

**Authors:** 


*A Treatise on Ruptures.* By JONATHAN F. C. H. MACREADY. Pp. xviii., 442. London: CHARLES GRIFFIN & COMPANY, LIMITED. 1893.

We have nothing but praise for this work, which gives a good summary of all that is at present known on the subject of Hernia. It is written in a modest manner, and makes no pretence of being an exhaustive work; but the author has brought together a large mass of material which he has well arranged and summarised, so as to give a readable and handy book of reference. The work is arranged in two parts: one treating of ruptures where the functions of the bowels are undisturbed, and the other where those functions are interrupted. It is illustrated by reproductions from photographs from nature, and many of these are of the most beautiful character, giving quite a life-like representation of the parts affected. The bibliographical notices are placed at the end of each chapter, a plan which is very convenient on the whole, although it naturally necessitates some repetition. The statistical tables seem very complete, and are largely drawn from the City of London Truss Society, to which the author is surgeon. His experience here has afforded him very valuable knowledge in the application of trusses, and one of the chief merits of this book is the description of the various kinds of instruments, illustrated by photographs showing them in position as worn.

The chapters on congenital hernia and on the predisposing causes of hernia are well and carefully written, and contain all that has been lately made known on these subjects. We are glad to see that the author rejects as apocryphal the theory that the testis may come down the crural canal and bring with it a hernia. One such supposed case was recently brought before a local society, but would not stand criticism. The operation of herniotomy is well and fully dealt with, and a chapter is devoted to the treatment of lesions of the bowel.

The author's remarks on the proper method to be pursued in cases of gangrene of the bowel are eminently judicious. He cautiously states that "the rule of treatment in London at the present day is to form an artificial anus;" but he gives very fully the arguments for and against resection of the gut, and the description of the latter operation is good and plain, though brief. Perhaps the least satisfactory part of the book is the chapter on "The Radical Cure of Hernia." After a short description of the various subcutaneous operations formerly in vogue, the author points out that "the first operation under aseptic precautions for reducible rupture in this country is believed to have been performed by Mr. Steele, of Bristol, in May, 1873." Liverpool surgeons are in the habit of calling this "the Liverpool operation," and we are glad that attention has been called to the prior claim of Bristol. A very few lines are devoted to a description of three or four ways of operating for the radical cure by the open method, and a table is added showing the after-condition of patients visiting the Truss Society. Upon the whole the author speaks with doubtful praise of this operation, and seems to us to do it but scant justice. The book is wonderfully free from errors, and has an excellent index.

